# Ichthyol in Eczema

**Published:** 1901-05-11

**Authors:** 


### ICHTHYOL IN ECZEMA.

In a lecture delivered at the London Polyclinic, Mr. Malcolm Morris[1] dealt, among other things, with the eczema of the menopause. Of this he says there are two special forms; one an acute eczema of the head and face, and the other that very acute form of the disease which occurs about the anus and vulva. In regard to the first of these conditions, namely, eczema of the head and face, it occurs in women at the change of life. A spare woman at that time of life suddenly begins to flush in the face perhaps after taking a meal. Later the disorder becomes a little more acute; she gets an eczema of the scalp, and it spreads down all over her face. For that condition there is, says Mr. Morris, no drug or combination of drugs which is of such service, not only to the eczema but to the other nervous symptoms connected with it, as ichthyol. If used in the right way it is an invaluable drug, and this is one of the diseases in which it is of the greatest possible use. It is, however, a nasty-smelling drug; therefore every attempt should be made to disguise the odour, and if given in the form of a sugar-coated tabloid there is no difficulty on that score. It can be given in tabloids coated with keratin which does not dissolve until it gets into the intestine, the result being that the patient does not get eructations of the ichthyol. The dose should be 2½ grains after each meal. At the end of two or three days the dose should be increased to 5 grains, and then by steps to 10 grains. If the patient tastes it much the stomach has got more than it can bear, and the dose should be cut down a little. But the effect of the drug in clearing away the symptoms is very extraordinary. With regard to local treatment this form of eczema requires rather more active treatment at the menopause than is needed at any other time.

[1] Lancet, May 4.

